# Possible Diagnostic Improvement for Cutaneous Leishmaniasis: Is It Achievable?

**Published:** 2018-07

**Authors:** Yasaman Taslimi, Sima Rafati

**Affiliations:** Department of Immunotherapy and Leishmania Vaccine Research, Pasteur Institute of Iran, Tehran, Iran

The parasite, *Leishmania*, is the causative agent of the disease leishmaniasis and is highly endemic in 98 countries spread across the tropics, subtropics and Mediterranean Basin. It is a vector-borne disease transmitted by the bite of infected sand flies, and it appears in three clinical manifestations namely: cutaneous, mucocutaneous, and visceral leishmaniasis. It is known that at least 21 species are pathogenic to humans. To control the disease, there is no human vaccine, and the sole approach is through chemotherapy. Unfortunately, the available anti-leishmanial drugs are problematic due to their high toxicity, as well as increased parasite resistance. Therefore, making accurate diagnostic decisions for clinical treatment is highly important.

Old World cutaneous leishmaniasis (CL) is mostly caused by two *Leishmania* species, named as *L. major* and *L. tropica* that produce skin ulcers. It is also important to note that the symptoms of CL can be confused with other skin diseases. Similar clinical symptoms such as sarcoidosis, lupus vulgaris, leprosy, and bacterial ulcer may have common clinical signs with CL ulcer. Therefore, the diagnostic confirmation of the parasite is mandatory before starting an appropriate treatment strategy.

The lesions caused by *L. major* are typically self-healing; however, treatment is still necessary in order to minimize significant scarring and reduce the risk of relapse. Lesions from *L. tropica* infections are more difficult to resolve and last for a longer period.

## New hope for accurate, painless, and rapid sampling approach for CL diagnosis

**Fig. 1:**
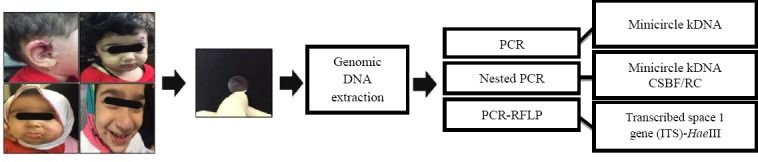
Sampling from sensitive-infected areas, especially in children, and the process of diagnostic determination for CL.

There is no point of care (POC) diagnostic test for CL, and it is highly crucial to develop immediate, convenient, and easy-to-use diagnostic test for this disease. To control the disease, it is vital to develop novel biomarkers, in order to design a more sensitive and specific diagnostic strategy. Most of the available and traditional tools are invasive and do not have enough sensitivity for CL diagnosis. There are several disadvantages of routine diagnostic tests such as the microscopic examination of smears and tissue scraping. Also, routine diagnostic tests are not only deficient in sensitivity but also cannot distinguish and identify the causing strains and their accuracy depends on the skill of health workers. There are more advanced approaches using PCR-based methods for species identification, which is specific, sensitive and a more reliable alternative tool. This approach usually requires the following samples: punch biopsies scrapings and lesion aspirate. Recently, non-invasive sampling has been developed by the use of tape strip for DNA isolation and species identification at different stages of disease, including acute, chronic and healed CL. Tape strip sampling is very easy and fast to perform, and there is no need to have special skills compared to lesion aspirate, biopsies, and scraping. This user-friendly approach is highly comfortable for children in endemic areas and also for sensitive parts of the body such as the lips, eyelid, ear, and face (Fig. 1). The main advantage of this strategy is that there is no risk of scarring and co-infection. It is also very cost-effective and highly applicable for remote-endemic areas.

## Possible future attempts for POC diagnostic tests for CL; Are they feasible?

The recommendation of World Health Organization (WHO) for any diagnostic tool in developing countries is to consider the sensitivity, specificity, accuracy, speed, accessibility, less facility requirements and cost. The current PCR-based methods for diagnosing leishmaniasis are highly sensitive and specific, but restricted to research laboratories, as well as referral hospitals with sophisticated equipments and expertise. Therefore, it is highly crucial to develop novel molecular methods that could be applied in resource-limited endemic areas. One of these breakthrough platforms is the loop-mediated isothermal amplification (LAMP) method, which is less expensive and does not require high-tech equipment like the PCR machine. This method can be utilized for the detection of any pathogenic microorganism with higher sensitivity and specificity than ordinary PCR. LAMP requires six different primers, two outer primers (F3 and B3), two inner primers (FIP and BIP), and two loop primers (LF and LB). There are only two options for enzyme with the best activity at 60 °C, *Bst* polymerase or *Bsm* polymerase that proceed under isothermal conditions. It produces a high amount of amplification product that ended up with simple visual detection. For the detection of LAMP product, there are different possibilities including extra Mg^++^ and fluorescent dyes. Higher specificity is also achievable by either targeting an internal sequence or by using a lateral flow dipstick format.

Altogether, with some modifications in LAMP, as a POC diagnostic platform, it is feasible to present a non-invasive diagnostic method with high sensitivity, specificity, as well as rapid and user-friendly application for the highly endemic disease, leishmaniasis.

## Diagnostic limitations in leishmaniasis

- There is no rapid and sensitive test for diagnosis of leishmaniasis.
- There is no approved non-invasive sampling method, especially for sensitive areas of the body and children.
- Current diagnostic methods are costly and require expensive equipment, electricity, and expert technician.

**More details in:**

1. ***Leishmaniasis worldwide and global estimates of its incidence*.** J Alvar et al. 2012. PloS one; Vol. **7**, pp. e35671.
2. ***Cutaneous leishmaniasis in Iran: Results from an epidemiological study in urban and rural provinces*.** F Norouzinezhad et al. 2016. Asian Pac J Trop Biomed; Vol. 6, pp. 614-619.
3. ***A novel non-invasive diagnostic sampling technique for cutaneous leishmaniasis*.** Y Taslimi et al. 2017. PLoS Negl Trop Dis; Vol. 11, pp. e0005750.

